# One-Pot Synthesis of Lamellar Fe-Cu Bimetal-Decorated Reduced Graphene Oxide and Its Enhanced Removal of Cr(VI) from Water

**DOI:** 10.3390/nano13202745

**Published:** 2023-10-11

**Authors:** Jing Li, Mingjie Fan, Ziting Yuan, Fang Liu, Miao Li

**Affiliations:** 1 Beijing Institute of Fashion Technology, Beijing 100029, China; lijing@bift.edu.cn; 2 School of Environment, Tsinghua University, Beijing 100084, China; liufang@imu.edu.cn; 3 Gudao Oil Production Plant, Shengli Oil Field, Dongying 257000, China; fanmj9741.slyt@sinopec.com; 4 Hebei Key Laboratory of Environment Monitoring and Protection of Geological Resources, Hebei Geo-Environment Monitoring Institute, Shijiazhuang 050022, China; isyuanziting@outlook.com; 5 School of Transportation, Inner Mongolia University, Hohhot 010021, China

**Keywords:** nano zero-valent iron (nZVI), reduced graphene oxide (rGO), Cu doping, hexavalent chromium (Cr(VI)), removal

## Abstract

Hexavalent chromium (Cr(VI)) is a typical heavy metal pollutant, making its removal from wastewater imperative. Although nanosized zero-valent iron (nZVI) and graphene-based materials are excellent remediation materials, they have drawbacks, such as agglomeration and being difficult to recycle. A facile synthesis method for decorating reduced graphene oxide (rGO) with ultrathin nZVI (within 10 nm) was explored in this study in order to develop an effective tool for Cr(VI) detoxication. Cu particles were doped in these composites for electron-transfer enhancement and were verified to improve the rate by 2.4~3.4 times. Batch experiments were conducted at different pHs, initial concentrations, ionic strengths, and humic acid (HA) concentrations. From these observations, it was found that the acid condition and appearance of Cu and rGO enhanced the treatment capacity. This procedure was fitted with a pseudo-second-order model, and the existence of NaCl and HA impeded it to some extent. Cr(VI) could be detoxified into Cr(III) and precipitated on the surface. Combining these analyses, a kinetics study, and the characterizations before and after the reaction, the removal mechanism of Cr(VI) was further discussed as a complex process involving adsorption, reduction, and precipitation. The maximum removal capacity of 156.25 mg g^−1^ occurred in the acid condition, providing a potential Cr(VI) remediation method.

## 1. Introduction

Chromium is a common element in the earth’s crust and is an important strategic metal resource. The chromium chemical, leather, and electroplating industries are the most important industries related to chromium [1], and it is irreplaceable in many commodities worldwide. However, chromium, with its hexavalent status, acts as a common hazardous contaminant. Cr(VI) is toxic to humans, causing dermatitis, bronchitis, liver damage, ulcer formation, and carcinogenic risk [2]. The improper mining of chromium ore and the chromium residue left by enterprises have caused serious pollution to water bodies and have even affected underground aquifers [3]. Some investigations have shown that the maximum concentration of Cr(Ⅵ) in groundwater can reach nearly 10,000 times higher than the maximum permissible limit in drinking water in China (0.05 mg L^−1^) [4].

Zero-valent iron (ZVI) has high reactivity and a high reduction capacity (φFe^2+^/Fe^0^ = −0.447 V), making it an excellent remover of a wide range of pollutants [5]. Owing to its advantages of low toxicity, low cost, easy operation, and low secondary pollution to the environment, its application in water remediation is receiving increasing attention. ZVI has been widely studied since Gillham and O’Hannesin first used ZVI for pollution control in 1994 [6]. Because the standard redox potential E^0^ of Cr(VI) ((Cr_2_O_7_^2−^/Cr^3+^) = 1.33 V) is positive and much higher than that of ZVI, Cr(VI) can easily be reduced to harmless Cr(III) with treatment by ZVI [7]. In particular, nanoscale ZVI possesses a much larger surface area than conventional ZVI, exhibiting much higher chemical reactivity. The research on Cr(VI) removal from water by ZVI began with the Permeable Reaction Wall (PRB) experiment conducted by Puls’ team in 1999 [8]. Then, Ponder et al. proved the advantage of nZVI in aqueous Cr(VI) pollution remediation over traditional iron powder/slags [9]. Despite its advantages, nano-zero-valent iron (nZVI), with its higher activity, still has obstacles in practical environmental remediation due to its defects of easy aggregation and passivation [10,11,12].

Various modification methods, such as metal doping, ferrite/sulfide modification, inorganic loading, and organic polymer stabilization and functionalization, have facilitated the improvement of nZVI [13]. Among these methods, loading in support matrices with high specific surface areas is the most popular and effective way to avoid aggregation and obtain dispersed nZVI. Graphene-based materials possess a series of excellent electrical, mechanical, optical, and chemical properties [14]. Moreover, graphene-based materials have received considerable attention due to their wider environmental applications [15]. They can act as adsorbents, photocatalysts, and sensors due to their excellent physical–chemical properties and high specific surface area (2630 m^2^ g^−1^, in theory) [15,16,17,18]. However, bare graphene materials are difficult to separate and recycle from environmental media, leading to waste and a risk of secondary pollution. Magnetic material decoration is usually used to facilitate graphene’s recycling. Through combining the advantages of nZVI and graphene, composite materials have gained increasing attention for the remediation of aqueous environmental pollution. Various organic and inorganic pollutants have been verified to be effectively removed by ZVI/graphene nanocomposites [19,20,21,22,23]. Graphene oxide (GO) is a versatile graphene-based material with abundant oxygen-containing groups, such as carboxyl, hydroxyl, and epoxy groups, which makes it easier to make surface modifications [24]. The application of GO enhances interactions with various contaminants from wastewater owing to its high surface area, hydrophilicity, efficient electron transformation, and adsorption capacity [24,25]. The combination of ZVI and GO was reported to exhibit excellent performance in dye degradation [26]. The synergy between ZVI and reduced GO showed a unique affinity for aqueous Hg^2+^ in Bao’s research [20].

Moreover, research has shown that a second metallic doping contributes to the formation of galvanic cells within ZVI composite materials, enhancing their pollutant removal capabilities. Precious metals are commonly used for metal doping in ZVI, showing high efficiency [27]. Despite their advantage, their cost limits their application. Copper, an essential trace element for life, has lower toxicity than other heavy metals. Copper is often used as a dopant metal in combination with iron [28]. It has also been proven to promote the removal of the organic compound 4-nitrophenol from water in the ZVI system. The optimal copper content is around 10 μmol Cu/g Fe, as excessive or insufficient amounts of copper have a detrimental effect on the system’s efficiency. The heterogeneous Cu layer on the surface of Fe/Cu bimetallic particles facilitates the degradation of pollutants, leading to a substantial improvement in the efficiency of the zero-valent iron system [29], whereas a uniform and denser Cu layer significantly reduces the reactivity. Moreover, Hosseini’s study demonstrated the enhanced migration capability of Fe/Cu bimetallic particles in sand columns, supporting their potential application for in situ groundwater remediation [30].

In order to balance the high reactivity and material stability, this study focused on the pathway to overcome agglomeration and improve activity through preparation at a small scale and with proper bimetal-doped modification.

In this study, graphene-based materials and metallic particles were integrated with zero-valent iron (ZVI) and achieved a uniform distribution of ZVI particles on the graphene layers. This process used a solvothermal reduction method as the one-pot synthesis approach, where high-valence iron, graphene oxide (GO), and Cu^2+^ ions were reduced simultaneously. With the aid of an aminosilane coupling agent, the ZVI particles were better connected to the reduced graphene oxide (rGO) layers, facilitating their combination. Through this procedure, ultrathin Fe/Cu bimetal particles decorating rGO layers were prepared. Then, these composites were characterized to identify their physical and chemical properties. The resulting composites were applied to treat water with Cr(VI). A series of factors, such as additional GO amounts, pH, ionic strength, humic acid (HA) concentration, and initial concentration, were investigated, along with an analysis of the removal mechanism. This research hoped to provide a promising one-pot fabrication method for enhanced Fe/Cu@rGO composites and develop further exploration of nZVI removal technology for heavy metals.

## 2. Materials and Methods

### 2.1. Experimental Reagents

Ferric trichloride (FeCl_3_, AR), sodium hydroxide (NaOH), hydrazine (N_2_H_4_·H_2_O, 98%), and (3-Aminopropyl)triethoxysilane (APTES) were purchased from Aladdin Industrial Co., Ltd. (Shanghai, China). GO (powder in a single layer) was purchased from Jiangsu Xianfeng Nanomaterials Technology Co., Ltd. (Nanjing, China). Cupric chloride (CuCl_2_, AR) was purchased from Sinopharm Chemical Reagent Beijing Co., Ltd. (Beijing, China). Humic acid (HA, CP) was purchased from Guangfu Chemical Reagent Company (Tianjin, China). Anhydrous ethanol and hydrochloric acid (HCl, 37%) were purchased from Beijing Chemical Reagent Company (Beijing, China). Deionized water was made in the laboratory and used for experiments.

### 2.2. Experimental Instruments

A digital-controlled magnetic agitator (CLT-1A, Youlian Instrument Research Institute, Jintan City, China), vacuum drying oven (Kewei; Beijing, China), and freeze dryer (FDU-1200, Tokyo Rikakikai Co., Ltd., Tokyo, Japan) were used for nanocomposite synthesis. A constant temperature water bath oscillator (THZ-82A, Jiangsu Science and Technology Instrument Co., Ltd., Nanjing, China) and UV–Vis spectrophotometer (DR6000, Hach Water Quality Analyzer (Shanghai) Co., Ltd., Shanghai, China) were used for the Cr(VI) removal batch experiments and detection. A transmission electron microscope (TEM; JEM-2100 LaB6, JEOL Ltd., Tokyo, Japan) and scanning electron microscope (SEM, Merlin Compact, Carl Zeiss AG, Jena, Germany), combined with energy-dispersive X-ray spectroscopy (EDS, X-Max^N^, Oxford Instruments Technology (Shanghai) Co., Ltd., Shanghai, China), were used to identify the morphology and composition of the nanocomposites. An X-ray photoelectron spectrometer (XPS, 250XL, Thermo Fisher Scientific Inc., Waltham, MA, USA) and X-ray diffractometer (XRD, D/max-2550, Bruker Corporation, Karlsruhe, Germany) were used to analyze the existence and valence states of the elements in the nanocomposites. A Raman spectrometer (inVia Reflex, Charfield, UK) was used to obtain the status of GO. Brunauer-Emmett-Teller (BET) surface areas were measured by N_2_ adsorption and desorption (Micromeritics ASAP 2046, Norcross, GA, USA). A vibrating sample magnetometer (VSM) (VSM, LakeShore7410; Westerville, OH, USA) was used for magnetic measurement.

### 2.3. Nanocomposite Fabrication

**Figure 1:**
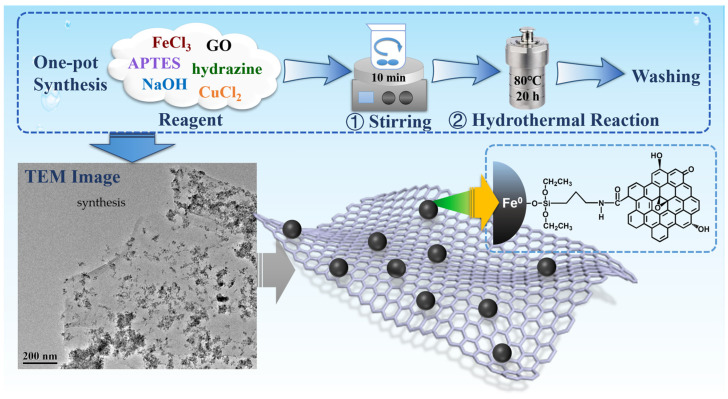
Material synthesis schematic.

In this study, the one-step solvothermal method for the synthesis of ZVI was modified to couple it with the reduction of GO (Fe@rGO) [23,31]. In the reaction, hydrazine acted as a reductant, reducing trivalent iron and graphene oxide simultaneously. In APTES, the Si-O bonds were connected to the iron particles, and the amino groups reacted with the carboxyl groups on the graphene oxide through an amidation process, forming a firm conjunction between nZVI and rGO. The specific experimental steps were as follows: A certain amount of GO powder was added to 12 mL anhydrous ethanol and subjected to low-temperature ultrasonication for 2 h for dispersal. Then, 1.5 g FeCl_3_, 0.2 mL APTES, 3.72 g NaOH, and 4.5 mL N_2_H_4_·H_2_O were added to the GO solution in sequence with constant stirring. Subsequently, the brown mixture was rapidly stirred for 10 min with a magnetic stirrer for homogenization and quickly transferred into a 50 mL stainless steel reaction vessel lined with polytetrafluoroethylene. The reaction vessel was placed in an 80 °C oven and maintained for 20 h. Then, the reaction vessel was opened after cooling to room temperature, and the black precipitate was collected through magnetic separation. The precipitate was washed three times with ethanol and deionized water, then dried overnight in a freeze dryer. The synthesis process is illustrated in Figure 1. The additive GO amount was tested in the experiments with gradients of 0, 0.05, 0.06, 0.07, and 0.08 g. In order to investigate the potential improvement with other metals, metal-doping modifications were performed with Cu [32,33]. The doping amount was set to be 10% of the molar quantity of iron based on previous research experience [33,34]. The resulting material was labeled as Fe/Cu@rGO.

### 2.4. Characterization

The synthesized nanocomposites were characterized with various instruments. TEM and SEM were employed for morphology observation, while EDS was used for elemental composition analysis. Raman spectroscopy was performed at an excitation wavelength of 532 nm to analyze the presence and graphitization state of graphene. XRD analysis was conducted to determine the material’s crystal structure, especially the existence of magnetic particles. The XRD spectra were acquired between 2θ of 10–90, with a step size of 0.05 and a 2 s dwell time. XPS was utilized to characterize the presence of individual elements in the sample, and the BET method was used to measure the material’s specific surface area. Magnetic analysis was carried out with VSM (−20,000~20,000 Oe) to determine the sample’s saturation magnetization. Additionally, the zeta potential test was employed to assess the surface charge of the material at different pH values.

### 2.5. Removal of Cr(VI) Experiment

#### 2.5.1. Batch Experiments

The 20 mg synthesized nanocomposites were placed in a 100 mL transparent glass bottle with the cap lined with aluminum foil. Then, 50 mL of Cr(VI) solution with a certain concentration was added to the bottle. After 10 s of ultrasonic dispersion, the bottle was placed in a constant-temperature shaker at 150 rpm at 25 °C for the reaction time. After the reaction, the supernatant was collected to determine the remaining concentration of Cr(VI). Blanket and parallel samples were set up for each group. Magnetic separation was performed to obtain the supernatant quickly, taking advantage of the magnetic material. The samples were filtered through a 0.22 μm membrane before determining the concentration if the material had lost its magnetism or the magnetic force was not strong enough for rapid separation.

#### 2.5.2. Cr(VI) Determination and Calculation

The Cr(VI) concentration was detected with a UV-vis spectrometer using the diphenylcarbazide spectrophotometric method, according to the Chinese national standard: Water quality-determination of chromium(Ⅵ)-1,5 Diphenylcarbohydrazide spectrophotometric method (GB 7467-87) [35]. This method is applicable to the determination of hexavalent chromium in surface water and industrial wastewater. The formed compound from the reaction of Cr(VI) and dibenzoyl dihydrazine had a purple-red color and could easily be determined with a UV–Vis spectrophotometer. The measurement was conducted at a wavelength of 540 nm in a quartz cuvette. The removal amount and removal efficiency were calculated based on the changes in the target substance’s concentration before and after the reaction, according to the following equations:

(1) $$Q_{t}=\frac{V\times \left(C_{0}-C_{t}\right)}{m}$$

Removal efficiency = (*C*_0_ − *C_t_*)/*C*_0_ × 100% (2)

In Equations (1) and (2), *Q_t_* represents the removal amount (mg g^−1^) of the target substance by the adsorbent material at time *t*, *C*_0_ is the initial concentration (mg L^−1^) of the target substance, *C_t_* is the concentration (mg L^−1^) of the target substance at time *t*, *V* is the volume (mL) of the solution, and m is the mass (g) of the added nanocomposites.

#### 2.5.3. Kinetic Experiment and Analysis

Graphene-based carbon materials are often reported to have outstanding adsorption capacity for heavy metals [15]. Pollution remediation by ZVI is also initiated by adsorption [36,37]. In our research, adsorption kinetics experiments over extended time periods (0~300 min) were conducted with a series of independent samples to gauge the adsorption rate and capacity of Fe/Cu@rGO towards Cr(VI) solution. The supernatant, with an initial concentration of 40 and 80 mg L^−1^, was determined by UV-vis after the reaction with 20 mg Fe/Cu@rGO at 25 °C. The experimental kinetic data were fitted to two commonly used kinetic models: the pseudo-first-order kinetic model (Equation (3)) and pseudo-second-order kinetic model (Equation (4)):

(3) $$\ln\left(q_{m}-q_{t}\right)=lnq_{m}-k_{1}t$$

(4) $$\frac{t}{q_{1}}=\frac{1}{k_{2}q_{m}}+\frac{1}{q_{m}}t$$

In the above equations, *t* (min) represents the reaction time. *k*_1_ (min^−1^) and *k*_2_ (g mg^−1^ min^−1^) are the rate constants of the above two models, respectively. *q_m_* and *q_t_* (mg g^−1^) are the maximum adsorption capacity and the adsorbed amount at time *t*, respectively.

## 3. Results and Discussion 

### 3.1. Characterization

**Figure 2:**
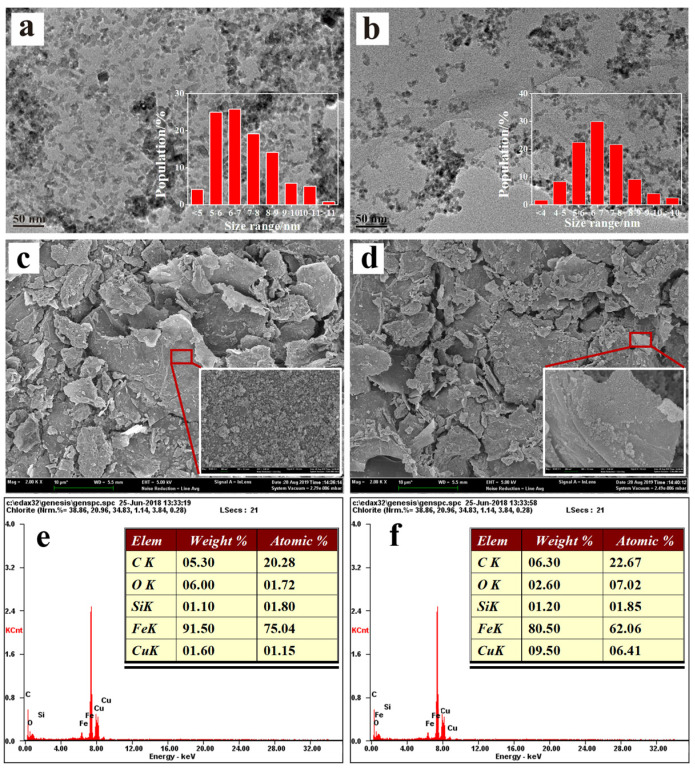
TEM (**a**), SEM (**c**) images, and EDS spectrum (**e**) of Fe@rGO; TEM (**b**), SEM (**d**) images, and EDS spectrum (**f**) of Fe/Cu@rGO (the insets in (**a**,**b**) show the size distribution of dark nZVI particles).

Microphotographs demonstrated the morphology of the nanomaterials, indicating their size, shape, and dispersibility. In both the TEM and SEM images (Figure 2), the materials showed granular dark nanoparticles dispersed on thin light layers. Based on empirical analysis, the transparent and low-contrast lamellar sheet was identified as an rGO sheet, while the dark particles were identified as iron particles. 

The size of the iron particles was approximately 4–10 nm, almost within 10 nm, which was much smaller than the conventional cubic ZVIs (>100 nm) synthesized through typical solvent thermal methods [38,39]. The rGO was primarily in a single-layer state, providing nZVI particles with an ideal supporter to avoid aggregation and further extension in size. The BET-specific surface area of Fe/Cu@rGO was measured to be 69.529 m^2^ g^−1^, which was significantly higher than that of conventional zero-valent iron particles with similar particle size synthesized using sodium borohydride liquid-phase reduction methods (approximately 35 m^2^ g^−1^) [40].

In the EDS spectra (Figure 2e,f), the material doped with Cu showed an obvious increase in the composition percentage of Cu compared with Fe@rGO, indicating successful doping. The ratio of Fe/Cu was about 10:1, in congruence with the addition ratio of the preparation. The ratio of O increased and Fe decreased with the addition of Cu, showing a slight oxidation trend with the effect of Cu. The existence of Si was also proved, suggesting a firm bond.

**Figure 3:**
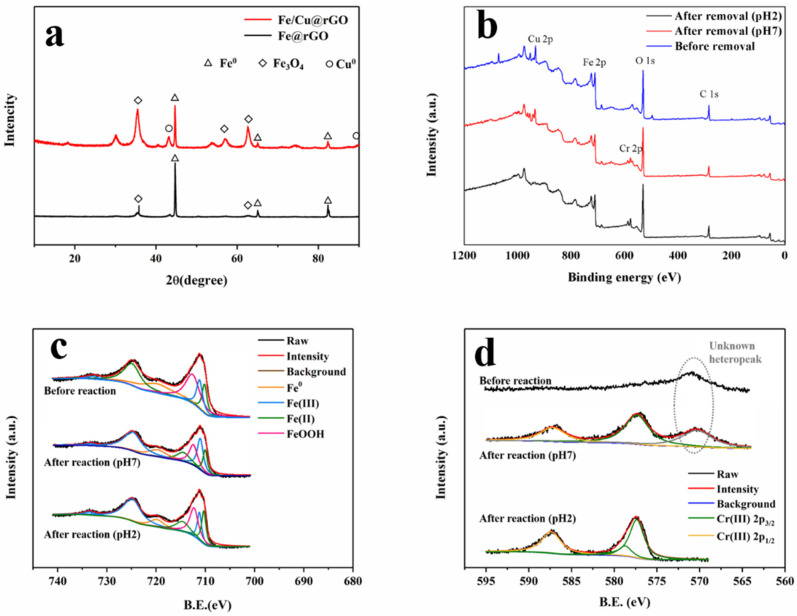
XRD spectra before the reaction (Fe@rGO, Fe/Cu@rGO) (**a**); the overview (**b**) and high-resolution XPS spectra for Fe (**c**) and Cr (**d**) of Fe/Cu@rGO (before and after the reaction).

In the XRD spectra (Figure 3a), the sharp peak at 44.7° and the small peaks at 65° and 83° were all characteristic peaks of the (110) crystal plane, indicating the presence of nZVI. The weaker diffraction peaks at 35.4°, 57.5°, and 62.5° suggested the existence of some iron oxides (Fe_3_O_4_), which was perhaps due to slight oxidation on the surface of the nZVI. The XRD diffraction peaks of Fe/Cu@rGO at 43.3° and 89.9° were in agreement with the phase of Cu^0^ [27].

The existence of Fe, O, C, and Cu was also confirmed by the XPS spectra (Figure 3b) and elemental content table (Table 1). In the XPS spectra (Figure 3b), characteristic peaks of Cr were observed in the material after the reaction, indicating the adsorption and deposition of chromium ions on the surface of the nanocomposite materials. In Figure 3d, except for the unknown peak at 570 eV (the impurity peak disappears in acidic conditions), distinct characteristic peaks of Cr(III) emerged after the reaction, while there was no detection of Cr(VI), even under neutral conditions. This suggested that perhaps all Cr(VI) adsorbed on Fe/Cu@rGO was completely reduced to trivalent chromium and deposited, which was consistent with previous research [41]. Figure 3c demonstrates the presence of zero-valent, divalent, and trivalent iron in the material before and after the reaction. The relative peak intensities indicate a higher proportion of zero-valent iron before the reaction. The decrease in surface iron content and the increase in oxygen content after the reaction also reflect this phenomenon, which was in agreement with the results from the EDS analysis. The XRD and XPS spectra before the reaction also proved the feasibility of the one-step synthesis method for Fe@rGO and Fe/Cu@rGO.

Overall, the results confirmed the successful synthesis of Fe@rGO and Fe/Cu@rGO composite materials and demonstrated their potential for Cr(VI) removal by efficient adsorption, reduction, and deposition of Cr(VI) to Cr(III) on the material surface.

**Figure 4:**
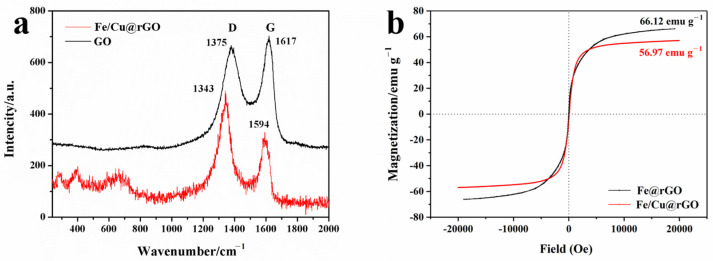
Raman spectra of Fe/Cu@rGO and GO (**a**) and VSM hysteresis curves (**b**) of Fe@rGO and Fe/Cu@rGO.

In the Raman spectra (Figure 4a), the peaks at around 1350 cm^−1^ and 1600 cm^−1^ corresponded to the D band and G band of graphene-like materials, respectively. The D band reflected the disorder-induced defect and lattice disorder in the material, and its peak intensity was related to the content of sp^3^-hybridized carbon. The G band reflected the symmetry and graphitization degree, and its intensity was related to the amount of sp^2^-hybridized carbon. From the Raman spectra, the characteristic peaks of Fe/Cu@rGO exhibited a blue shift compared with bare GO, which can be attributed to the reduction of graphene oxide and the interaction between the GO layer and iron particles. The ratio of G band intensity to D band intensity (ID/IG) is commonly used to evaluate the graphitization degree and defect level of carbon materials. In this study, the ID/IG value of the bare GO was 0.926, which increased to 1.458 after the solvent thermal reaction, indicating an increase in defects and sp^3^-hybridized carbon on the graphene sheets. This result was consistent with the findings of other researchers [42].

The addition of Cu did not cause significant changes in the saturation magnetization of the material, which were 66.12 emu g^−1^ and 56.97 emu g^−1^ before and after copper doping, respectively (Figure 4b). Combined with the XRD analysis, this indicated that the slight copper doping accelerated the oxidation of zero-valent iron (ZVI), resulting in a minor decrease in saturation magnetization. However, the overall influence on the magnetic properties of Fe/Cu@rGO can be neglected because of its excellent magnetic separation performance. Additionally, it was observed that these materials had very low coercivity, making them superparamagnetic materials, which facilitated rapid redispersion after magnetic separation.

### 3.2. Investigation of Factors Affecting Cr(VI) Removal

#### 3.2.1. The Effect of Cu Addition and rGO Amount

**Figure 5:**
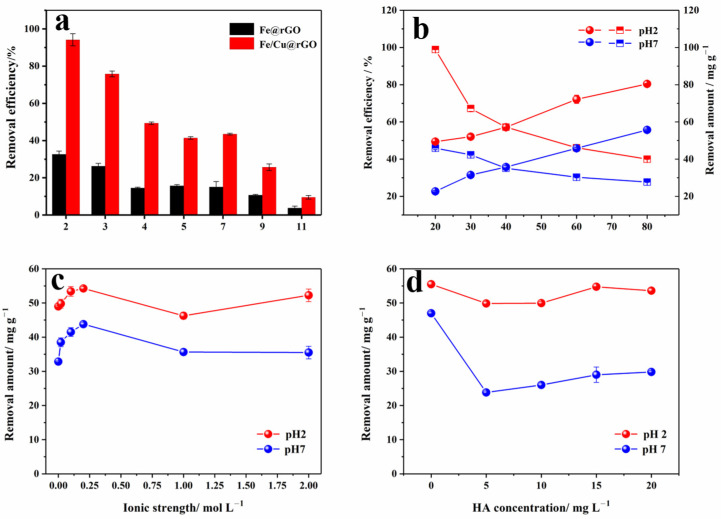
(**a**) The influence of pH value, (**b**) initial concentration, (**c**) ionic strength, and (**d**) HA concentration of the solution on the removal performance.

The addition of Cu enhanced the treatment capacity significantly (Figure 5a). In the pH range of 2 to 11, the Cr(VI) removal efficiencies of Fe/Cu@rGO reached about 2.4~3.4 times of those without Cu. This phenomenon may have been induced by the enhanced galvanic effect or electrochemical effect formed between ZVI and the added Cu^0^, and ZVI and Fe/Cu could serve as electron donors in the treatment of chromium [34,43].

The GO additive amount was also investigated in the range of 0 to 0.08 g. The modification of Cu and rGO facilitated Cr(VI) removal. The highest efficiency occurred when 0.05 g of GO was added to the fabrication system (Figure S1). In addition, the more GO added, the more difficult it was for the composite materials to be separated during the experiment. This may have been due to the magnetic hindrance of excess nonmagnetic GO. The Fe/Cu@rGO-0.05 materials were selected to be adsorbents in subsequent experiments and denoted as Fe/Cu@rGO.

#### 3.2.2. The Effect of pH 

Based on Figure 5a, the influence of aqueous pH on Cr(VI) removal by materials with and without Cu doping was examined. The observed influencing trend of pH was consistent with the results from the previous research [44]; lower pH promoted the reaction. For the removal efficiency of Fe/Cu@rGO, there was an increase of 10 times with the decrease of pH from 11 to 2. The highest removal efficiency was found to be 94.17%. As for Fe@rGO, the increase was about eight times. Graphene-like materials and ZVI materials usually hold positive surface charges in acidic solutions, which become negatively charged. The isoelectric point of ZVI is about pH 7–8, and it is positive below 7 [45]. On the other hand, Cr(VI) primarily exists as the monovalent anion HCrO_4_^−^ under acidic conditions (<pH 6) and as the divalent anion CrO_4_^2−^ under alkaline conditions. Therefore, the acidic conditions favored the electrostatic adsorption of Cr(VI) onto the material surface.

#### 3.2.3. The Effect of Initial Concentration

In our experiment, the initial concentration of Cr(VI) was also investigated (Figure 5b). Each adsorbent has its maximum removal capacity or an inflection point if the primary removal mechanism is adsorption. As shown in Figure 5b, the removal capacity exhibited a stable growth in the range of 20~80 mg L^−1^ (with a solution volume of 50 mL and Fe/Cu@rGO amount of 20 mg), and there was no clear indication of saturation. Nevertheless, the removal efficiency significantly decreased after 20 mg L^−1^ under pH 2 conditions, with an efficiency of 99% and a residual concentration of 0.23 mg L^−1^. Therefore, the removal process may not be promoted by only electrostatic adsorption.

#### 3.2.4. The Effect of Ionic Strength

Real water samples typically contain impurities. The ionic strength is a physicochemical parameter commonly observed in actual water samples, and it can also influence the activity of ZVI. In this experiment, NaCl was used to adjust the ionic strength in a wide range of 0~2 mol L^−1^, which is common in natural environments. As shown in Figure 5c, Cr(VI) removal by the Fe/Cu@rGO material fluctuated with the increase in NaCl concentration. The removal efficiency increased by 10.7% and 33.3% at both pH 2 and pH 7, respectively, before 1 mol L^−1^. After a gentle drop at 1 mol L^−1^, it increased again but was still lower than prior. The presence of an ionic solution has a corrosive effect on ZVI, which promoted ZVI’s participation in the reaction, while the Cl^−1^ may have demonstrated a competitive relationship with Cr(VI) ions. All in all, the presence of NaCl enhanced the Cr(VI) removal reaction, especially around 0.2 mol L^−1^.

The removal performance was also tested in two tap water samples and with deionized water (DI water) for contrast. Tap water 1# was collected outdoors in Caoqiao Village in Beijing, which was low quality and reported to have scales after boiling by the residents. Tap water 2# was collected indoors in Zhongwu Village in Beijing, which was of higher quality. The typical ions in these two samples are listed in Table S1. The ionic strength was obviously in accordance with the observed quality. The removal efficiency of added Cr(VI) at pH 7 was in the following sequence: DI water > Tap water 2# > Tap water 1#, indicating the water quality, particularly the ionic strength, had an important impact on Cr(VI) removal. The higher the ionic concentration, the lower the removal performance of Fe/Cu@rGO for Cr(VI). This phenomenon was perhaps due to the competition and by-products formed by the ions and nZVI [46,47].

#### 3.2.5. The Effect of HA

Humic acid (HA) is another common component in real water [48]. To simulate actual water conditions, the influence of HA on Cr(VI) removal was investigated. As shown in Figure 5d, when the HA concentration increased from 0 to 20 mg L^−1^, the maximum decreases in Cr(VI) removal at 5 mg L^−1^ were 10.1% and 49.6% at pH 2 and pH 7, respectively. Subsequently, along with the increase in HA concentration, the removal capacity slightly increased but was far from the level obtained in treatments without HA. This suggests that the presence of HA competes with Cr(VI) for adsorption and then provides new adsorption sites for chromium as the concentration increases.

### 3.3. Kinetic Analysis

**Figure 6:**
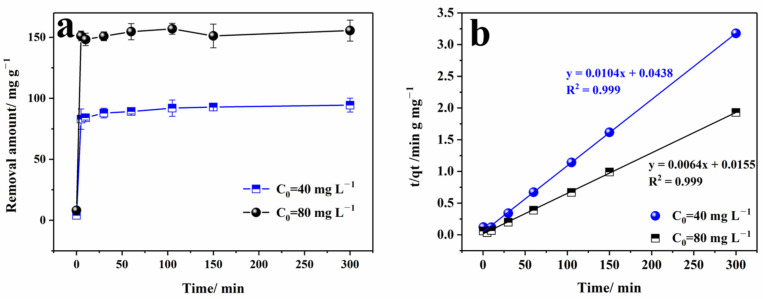
Kinetics curves of Cr(VI) removal by Fe/Cu@rGO materials (**a**); the fitted curve of quasi-second-order dynamics (**b**).

Kinetics experiments can reveal the determination step of adsorption rates. The experiments were conducted at 25 °C and pH 2, with initial concentrations of Cr(VI) set at two levels of 40 mg L^−1^ and 80 mg L^−1^ in 50 mL of solution. The material dosage was 20 mg. As shown in Figure 6a, the reaction proceeded quickly in the first 5 min, and the removal rate changed gradually with a slight increase over time thereafter. At 5 min, the removal efficiencies reached above 82% and 75% for 40 and 80 mg L^−1^, respectively. At this time, all the active sites were available, and the removal rate was rapid. At the end of the interval, the efficiencies were maintained at around 95% and 80%, respectively. During this period, the active sites were occupied by Cr (VI) ions, and the removal rate slowed down. The fitted result in Figure 6b indicated that reactions at both the Cr(VI) concentration levels followed the quasi-second-order kinetic equation, with an R^2^ of 0.999, which was similar to Waghmare’s study with polypyrrole/GO-modified ZVI nanoparticles [49]. In contrast, a similar Cr(VI) removal study using GO/rGO-base composite materials without ZVI followed the pseudo-first-order reaction [26]. This confirmed the chemical-reactive function of ZVI. The calculated maximum removal capacities were 96.15 mg g^−1^ (40 mg L^−1^) and 156.25 mg g^−1^ (80 mg L^−1^), which were in agreement with the actual experimental values. The study of the pseudo-second-order kinetic model indicated that the chemical adsorption process was the rate-determining step of the reaction, consistent with the analysis results from XPS.

## 4. Conclusions

This study explored a one-step synthesis method for Fe/Cu@rGO nanocomposites and investigated the effect of Cu doping and rGO addition on the removal of Cr(VI), as well as the influence of environmental conditions, such as pH value, initial Cr(VI) concentration, ionic strength, and humic acid concentration, on the Cr(VI) removal efficiency. The characteristics of nanocomposite materials were verified, and the removal mechanism was discussed considering the material properties before and after the reaction, the influence of the environment, and the reaction kinetics.

Fe/Cu@rGO was successfully prepared with the solvent-thermal method. During this process, the reduction of iron salts to ZVI occurred, along with the reduction of Cu salts and graphene oxide. The material exhibited a layered structure resembling graphene, with ultra-small ZVI particles dispersed on the layers. This contributed to enhanced adsorption capacity and improved reactivity due to the excellent dispersion of nZVI particles and electron transfer ability. Doping with a small amount of Cu improved the outcome.

The removal of Cr(VI) by Fe/Cu@rGO is a complex process involving adsorption, reduction, and precipitation. The removal of Cr(VI) is influenced by environmental conditions such as pH, ionic strength, and humic acid concentration. The kinetic analysis showed that the maximum removal capacities were 96.15 mg g^−1^ (C_0_: 40 mg L^−1^) and 156.25 mg g^−1^ (C_0_: 80 mg L^−1^) in an acidic environment, and the kinetic behavior followed the pseudo-second-order equation, indicating chemical adsorption. The rate of this adsorption process was related to the squared number of unoccupied sites on the material surface. The fewer unoccupied sites, the lower the adsorption rate.

## Figures and Tables

**Table 1:** Element composition before and after the reaction (from XPS).

Material	Atomic Percentage/%				
	Fe	Cr	O	C	Cu
Fe/Cu@rGO before reaction	19	2.36	39.73	34.72	4.19
Fe/Cu@rGO (pH 2) after reaction	16.81	3.73	47.84	30.37	1.25
Fe/Cu@rGO (pH 7) after reaction	15.78	5.02	44.06	28.96	6.19

## Data Availability

Data sharing is not applicable to this article.

## Supplementary Materials

The following supporting information can be downloaded at: https://www.mdpi.com/article/10.3390/nano13202745/s1, Figure S1: The influence of GO amount on the removal performance. Figure S2: The removal performance by Fe/Cu@rGO in different water samples. Table S1: Typical ionic concentration in different water samples.
