# Supplementary material for: One-Pot Synthesis of Lamellar Fe-Cu Bimetal-Decorated Reduced Graphene Oxide and Its Enhanced Removal of Cr(VI) from Water
Source: Nanomaterials (Basel). 2023 Oct 11;13(20):2745. doi: 10.3390/nano13202745 (PMC10608891; doi:10.3390/nano13202745)
Supplement: Supplementary file 1 [file nanomaterials-13-02745-s001.zip › nanomaterials-2600944-supplementary.pdf]

Supplementary materials

One-Pot Synthesis of Lamellar Fe-Cu Bimetal-Decorated Reduced Graphene Oxide and Its Enhanced Removal of Cr(VI) from Water

Jing Li[1,2], Mingjie Fan[3], Ziting Yuan[4], Fang Liu[2,5], Miao Li[2*]

[1] Beijing Institute of Fashion Technology, Beijing 100029, China; lijing@bift.edu.cn
[2] School of Environment, Tsinghua University, Beijing 100084, China; liufang@imu.edu.cn
[3] Gudao Oil Production Plant, Shengli Oil Field, Dongying 257000, China; fanmj9741.slyt@sinopec.com
[4] Hebei Key Laboratory of Environment Monitoring and Protection of Geological Resources, Hebei Geo-Environment Monitoring Institute, Shijiazhuang 050022, China; isyuanziting@outlook.com
[5] School of Transportation, Inner Mongolia University, Hohhot 010021, China
* Correspondence: miaoli@tsinghua.edu.cn

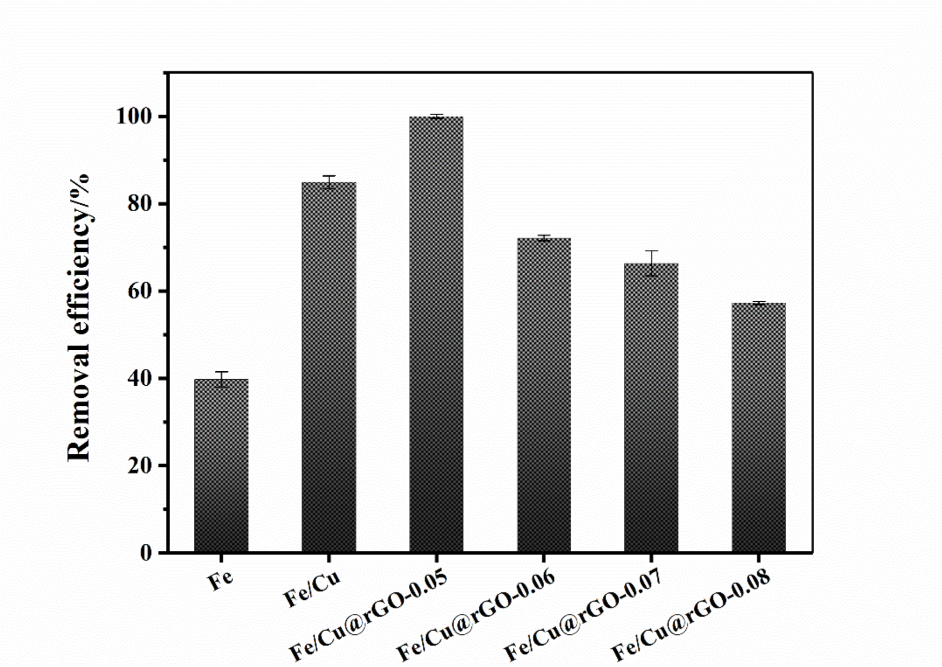

Figure S1. The influence of GO amount on the removal performance of Cr(VI). (pH=2, Cr(VI) initial concentration: 40 mg L^{-1}, adsorbent concentration: 0.4g L^{-1}).

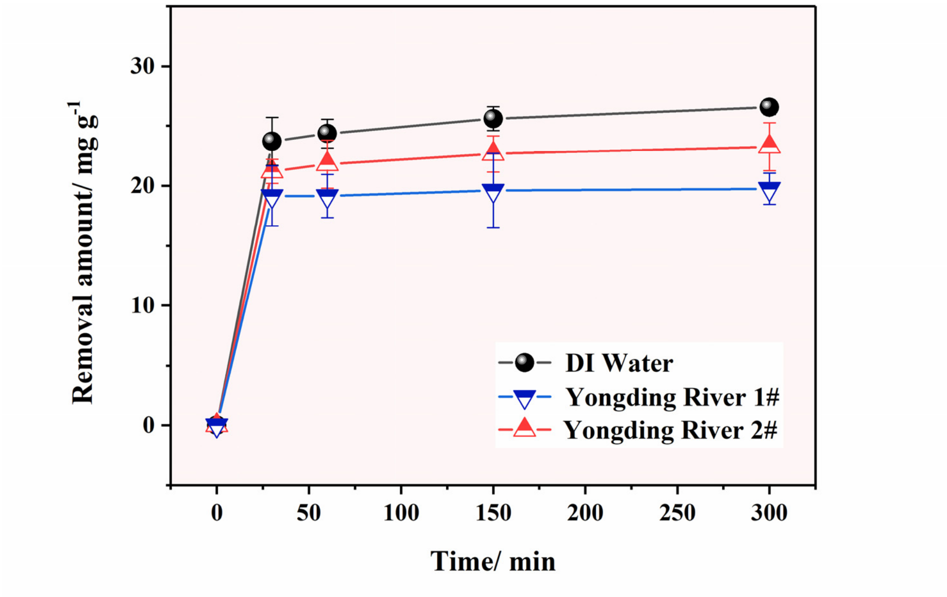

Figure S2. The Cr(VI) removal performance by Fe/Cu@rGO in different water samples.

Table S1. The typical ionic concentration in different water samples.

Water Sample	Ionic concentration/mg L^{-1}				
	Na^+	Cl^-	SO_4^{2-}	NO_3^-	HCO_3^-
DI water	-	-	-	-	-
Tap Water 1#	135	162.1561	217.8186	141.0428	586.64
Tap Water 2#	28.3	27.5731	61.8122	5.3029	259.2
